# Curious Games: Game Making, Hacking and Jamming as Critical Practice

**DOI:** 10.3390/bs15101415

**Published:** 2025-10-17

**Authors:** Chloé Germaine, Paul Wake

**Affiliations:** School of English, Manchester Metropolitan University, Manchester M15 6BH, UK; c.germaine@mmu.ac.uk

**Keywords:** creative methods, higher education, research methods, critical design, critical literacy, game design, hacking, playful learning

## Abstract

In this article we establish the affordances of game making, hacking, and jamming as critical practices in teaching and research. We explain the origins of our approach in specific teaching and research projects and consider their impact on our scholarly practice. First, we interrogate the value of game making through a project in which students at the Manchester School of Architecture were tasked with exploring questions relating to Britain’s post-war power infrastructures through the creation of games (in place of traditional essays). These games were subsequently used to share research with the public. Second, we develop the concept of game hacking in relation to our own research practice, where we have used it to creatively investigate designing for sustainability and as a practice for imagining alternative climate futures. Finally, we move from game hacking to a consideration of jamming through reflections on a participatory research project with young people, which sought to understand how board game play could support their climate action. There, game hacking became an anarchic process that enabled young people to interrogate the world and develop critical frameworks for speaking out about their experiences. Using game making in the HE classroom led us to employ hacking as a research method, which in turn prepared us to recognise and value the anarchic jamming that emerged in our participatory project with young people. That jamming experience has subsequently transformed how we approach both teaching and research, making us more attentive to moments when we might be willing to dwell in apparent unproductivity.

## 1. Introduction

### 1.1. Overview

Research on game-based learning has established that games are useful interventions in education, promoting improved outcomes from primary school to universities. In this article we accept the positive correlation between games and learning outcomes. However, our focus is on non-didactic interventions, on the potential of games to prompt critical thinking, for them to disrupt knowledge hierarchies and facilitate the sharing of knowledge and experience in multiple directions in pursuit of transformative research. This involves the democratisation of teaching and research (in the tradition of radical pedagogies) in such a way that teachers and students (as well as researchers and civil society), are equal partners in the creation of knowledge pursued with the aim of improving social conditions (Freire, 2017). To engage in this kind of work with games, we must attend to how we figure games and play in research, moving beyond the use of games as educational tools that address perceived deficits in their players, or as tools through which to extract data from participants. Here we propose game making, hacking and jamming as critical practices for researchers who would cultivate what Carr and Kemmis (1986, p. 162) describe as ‘a form of self-reflective inquiry’, those who wish to develop their own practices and those of the institutions in which research is undertaken. The researcher as hacker collaborates with all those involved in the coproduction of knowledge, understanding this as a relational and dialogic process, and engaging in ongoing reflection on the purpose and value of research. Building on the positive findings from existing research on game-based learning but looking to pedagogies and practices beyond its current scope, we propose hacking and jamming as interventions that promote and foster critical literacy in both teaching and research within Higher Education.

We use the term critical literacy in the sense developed through social justice pedagogies (Freire & Macedo, 1987; Knoblauch, 1990; Freire, 2017), following Shor’s (1999) account of critical literacy as the capacity to question power relations, dominant discourses, and to discover alternative paths for self and social development. We chart the development of game hacking and jamming through our teaching and research practice, showing how they originate in our use of game making as a learning and assessment tool in the university classroom, and how they subsequently evolved through our participatory action research (Fals Borda, 1987, 1988, 2001; Fals Borda & Rahman, 1981), a research methodology that shares a philosophical lineage with critical literacy in that it works with participants as co-creators of research in a process of inculcating consciousness of social conditions. As we progress through our case studies, we chart the movement from game play (as a learning activity) through game making (as a more active form of the remediation of knowledge) to hacking and, finally, to jamming as a disruptive learning experience and research tactic.

Games are routinely used in educational settings and there is a wealth of literature on the benefits of game-based learning (Aprea & Ifenthaler, 2021; Hartt et al., 2020; Pan et al., 2024; Rye et al., 2025; Su & Zou, 2024). We acknowledge this work but focus on how games intersect with creative and action research methods, addressing teachers and researchers in university contexts where research and learning are entangled. Making, hacking and jamming games compose a critical practice that has much in common with established methods of engaged research, such as participatory, action, and co-creative methods. However, it is also distinct from these because it is grounded in the unique affordances of games themselves and is therefore able to offer alternative approaches in the posing, exploring and solving of research questions and problems. As educators already know, games are more than consumer products or objects of leisure. However, they also have more potential in education than use as didactic teaching tools. Their potential also exceeds their typical use as tools to facilitate participation by stakeholders (such as a means for the collection of empirical data) or as communicative research outputs aimed at the general public, which is how they are typically leveraged within academic research (Houghton, 2022; Iacovides et al., 2019; Ouariachi et al., 2019; Squire, 2021). Making, hacking and jamming is about doing research with games in a more collaborative sense than these existing practices.

### 1.2. Structure and Key Terms

This article charts the evolution of our practice through distinct but related approaches. We begin with game making, a process whereby students create games to explore their learning and to communicate research questions studied on their courses. Game making is a way of remediating knowledge into playful forms. From this we develop game hacking, a critical–creative research practice that interrogates and transforms existing games to reveal the ideologies embedded in their systems and mechanics. Hacking, as we conceive it, is both a mode of reading the world, by analysing how games model and naturalise worldviews, and a means of speaking back to it, making visible what ideologies or systems obscure or take for granted. Hacking is therefore a collaborative research practice that leverages games in ways that enable participants to name and challenge the structures that shape their lives. Finally, we arrive at game jamming, an anarchic and reflexive process that disrupts educational and research hierarchies, and that resists outcome-oriented agendas, instead privileging a process of collective inquiry. These three modes (making, hacking, and jamming) represent an intensification of critical engagement, moving from the communication of learning through an orderly process of game making, to enabling critical research via disrupting game mechanics and stories, to the upturning of expected outcomes of research, and the privileging of slow, collaborative meaning-making.

We trace this progression through three case studies drawn from our teaching and research practice. The first examines game making as pedagogy in an architecture course on post-war infrastructure. The second explores our development of hacking as a research method, exemplified through our transformation of the nature-themed board game *Photosynthesis* (2017). The third details our participatory action research with young people on climate action, where our planned hacking sessions evolved into something more uncontainable—what we came to call jamming (see Table 1).

## 2. Materials and Methods

### 2.1. The Affordances of Games for Teaching, Learning and Research

Our practice is rooted in the unique affordances of games themselves which are, according to Mäyra (2009, p. 313), ‘multi-layered systems and processes of signification that mix representational and performative, rule-based and improvisational modes.’ It is precisely because of this mix of the rule-based and improvisational, the representational and performative, and the fact that one cannot study games without players nor vice versa, that game studies have the potential to be a ‘radical, transformative mode of scholarly practice’ (Mäyra, 2009, p. 313). To our thinking, games are processes and events (not merely narrative texts, cultural objects, nor teaching tools). Elsewhere, we highlight the ‘entangled’ nature of games, by which we mean that each game is a unique performance, or phenomena, arising from the interplay of components, rules, social contexts, and more, and argue that they continually throw up ethical questions about the ‘cuts’ of inclusion and exclusion and shifting and provisional power structures that emerge through play (Germaine & Wake, 2022; Wake, 2022). Games are participatory in nature, bringing together players, components and environments in dynamic networks of play that are highly contingent and open to transformation.

Games also hold a persuasive power, variously described in terms of ‘simulation’ (Frasca, 2004), ‘procedural rhetoric’ (Bogost, 2007) and ‘expressive processing’ (Wardrip-Fruin, 2009). As Bogost (2007, p. vii) has noted, games invite players to interact with systems and form judgements about them. In other words, they provide designers and players with an opportunity to both mount and critique arguments, to test hypotheses, to collaborate, to compete, to succeed, and fail. Defined by uncertainty of outcome (Elias et al., 2012, p. 137), games are speculative: their rules ‘push players to explore previously uncharted possibility spaces. They *unleash creativity* and *foster strategic thinking*’ (McGonigal, 2012, p. 21). ‘Half-real’ (Juul, 2005), but not separate from reality (Germaine, 2022b, 2025; Zimmerman, 2012), games promote the imagining of alternative worlds and ways of thinking and so provide an apt companion for pedagogies rooted in social justice and critical literacy, as well as critically engaged forms of action research. This claim for the radical potential of games in learning and research is not hindered by their rule-based nature. Indeed, it is our contention that rules promote rather than stifle creativity, especially when the constraints or limits imposed by rules become the focus of activity. Because games, and analogue games in particular, make plain their rules and mechanics, they invite modes of counter play, such as game hacking (Germaine & Wake, 2024), which subject the rules of the game (and the systems they model) to critique and transformation.

Mary Flanagan articulates this potential of games in *Critical Play* in which she asks, ‘what if some games, and the more general concept of “play,” not only provide outlets for entertainment but also function as a means for creative expression, as instruments for conceptual thinking, or as tools to help examine or work through social issues?’ (Flanagan, 2009, p. 1). In the decade and half since the publication of *Critical Play*, this ‘what if’ has been partially realised in the increasing use of games in education (Brown, 2014; Cai et al., 2017; Su & Zou, 2024; Whitton, 2009, 2014); in communicating ideas and values (Flanagan & Nissenbaum, 2014; Gislam et al., 2025; Illingworth & Wake, 2019; Jerrett & Howell, 2022); in promoting activism (Chess, 2020; Glas et al., 2019; Vervoort et al., 2024); in creative methods where games have been useful in collecting data (Gauntlett, 2007; Kara, 2020); in the reading of games as cultural texts (Bostan, 2021; Cole & Barker, 2020; Shaw, 2025); and in promoting the impact of academic work, or generating social impact in other ways (Grace, 2020; Houghton, 2022; Squire, 2021). These mobilisations of games in pursuit of pre-defined outcomes notwithstanding, the potential of game design as a mode of conceptual thinking in relation to the critical literacy and other forms of radical pedagogies remains to be realised. As Coulton and Hook (2017) argue, game design research, which considers games themselves as objects of enquiry, would benefit in moving towards approaches favoured in *design-led* research, especially those of critical and radical design. Our research praxis takes up this challenge, noting that it entails considering the process of game design itself as a mode of creative research, one that (as in critical design) might open ‘the unavoidable plurality of the future’ (Coulton & Hook, 2017, p. 116).

Our use of games in research and learning within Higher Education thus also takes inspiration from critical design practice which proposes that product and industrial design, rather than being limited to the production of objects for fiscal gain and technological development, ‘can be used to mobilize debate and inquire into matters of concern through the creative processes involved when designing objects’ (Malpass, 2017, p. 1). This shift in design practice, which Matt Malpass describes as being *affective* rather than *explanatory*, an opening up of lines of inquiry rather than providing answers or solutions, is something our research praxis explores, capitalising on the performative and entangled nature of games which, on an ontological level, open to the possibility of radically different outcomes. Games are, we contend, a perfect mode for undertaking what Illich (1973) describes as ‘counterfoil’ research, research that breaks with a growth-oriented agenda, and instead seeks to examine the tools, structures, relationships and systems that drive injustice and crises. In this way, our use of games in both teaching and research contribute to practices across education aimed at fostering critical literacy and challenging hegemonic discourses and hierarchies of power that structure education and research. This is why it is important to move from using games as teaching tools to (re)making and hacking games, modes of learning and play that are interrogative, curious, supporting collective inquiry, reflection and action.

### 2.2. Game Design as Pedagogy and Research Practice in Higher Education

There are two key strands in the literature on games and learning which, broadly speaking can be characterised as being concerned with (1) the use of games and/or play in the classroom, especially the use of “serious” or educational games for teaching (Aprea & Ifenthaler, 2021; Brandl & Schrader, 2024; Brown, 2014; Cai et al., 2017; De Gloria et al., 2014; Hartt et al., 2020; Houghton, 2022; Kafai & Burke, 2016; Pan et al., 2024; Schrader, 2022; Su & Zou, 2024; Whitton, 2009), and (2) the gamification of education (Araya et al., 2019; Christopoulos & Mystakidis, 2023; Dichev & Dicheva, 2017; Khoshnoodifar et al., 2023; Li et al., 2023). The model we outline in this essay explores a third, less studied model: the integration of game design in Higher Education teaching and learning *as a research process and critical pedagogy*.

The use of game design in Higher Education, outside courses on game design itself, has been relatively limited. One notable exception to this is Philip Sabin’s work at King’s College London which embeds game design in the teaching of military strategy and history. Sabin’s work provides useful insight into the affordances of game design as pedagogical method. Four of the potential benefits of game design as learning set out by Sabin are:

- The requirement that users and designers engage systematically with the underlying dynamics of situations.
- The engagement of users and designers in an active learning process through the engagement with the decision elements of historical, or indeed hypothetical future, scenarios.
- The improvement of feedback, for both instructors and students, as to the level of understanding of the user and player’s understanding of the situations modelled.
- They provide broader transferrable skills, including teamwork, design skills, and the dissemination of ideas.

Sabin’s approach leverages those affordances of games already identified by scholars (discussed above) who argue for their role in promoting systems thinking, speculative or hypothetical thinking, and generating engagement through active learning.

This pedagogical model of learning through making games can also be found in the work of Kafai and Burke (2016), where coding skills and video game making are proposed as a participatory pedagogy. Here the focus is not on Higher Education, but on how coding video games fosters active learning and supports the development of literacy across different educational settings. Despite their different contexts, the work of Sabin, Kafai and Burke suggests a shift from the use of games as tools for instruction in education, to the making of games for participation in learning, what Kafai and Burke call “constructionist gaming”. While Sabin argues that game making focuses students on recreating and modelling real-world historical situations with high levels of accuracy, for Kafai and Burke, the focus is less on simulation through game making, and more on unleashing students’ creativity.

In our own teaching and learning practice, game making is focused on developing critical thinking, and the games produced explore hypotheses and/or embody research processes and questions. They are perhaps better thought of as thought experiments in which the ‘what if’ aspect is placed at the foreground. In this work, our game making teaching and learning practice focuses on game mechanics and dynamics, rather than on their role as narrative or cultural texts. Our practice begins with Chris Crawford’s well-known definition of games in *The Art of Computer Game Design* (Crawford, 1984), which identifies four common factors: representation, interaction, conflict, and safety. Crawford’s definition begins with the idea of representation, suggesting that, through the combination of ‘explicit rules’ (its formal system) and the creation of a ‘model world,’ games represent a subset of reality. This concept of representation through rules is key to our understanding of the potential of games for teaching, learning and research in Higher Education, since playing *with* the rules, as in our practice of game hacking described below, requires that Crawford’s concept of interaction extends to the ability to see through, and tinker with, the formal game system.

This is not to say that games are only formal, mechanical systems. As Raph Koster notes, players ‘see through the fiction to the underlying mechanics, but that does not mean the fiction is unimportant’ (Koster, 2014, p. 166). While Koster privileges mechanics over fiction, seeing the latter as a ‘skin’ that is overlayered onto formal game systems, we see the double vision of the player as a key affordance of games as critical practice in teaching, learning and research. For games to foster critical literacy, it is necessary for mechanics and fiction to be visible simultaneously: the relative transparency of the fictional ‘skin’ allowing players to map connections between the two, opening the potential for games to invite critique and reflection. While game systems are often seen to precede game fictions, in games that represent aspects of reality, the extreme form of which is found in simulations like those explored by Sabin and his students, the reality represented (which might be a hypothesis, or, indeed, a fiction) necessarily precedes the mechanics. The role of the player—‘interaction’ in Crawford’s terminology—in this process is crucial. Players are encouraged to explore the causal relationships from a range of angles, focusing on the underlying dynamics of given situations rather than on establishing fixed (historical) narrative accounts of specific historical outcomes. For Gonzalo Frasca, this shift away from fixed sequences of events, and the incorporation of behavioural rules is a key feature of games which, while sharing common elements with other forms of representation, offer ‘distinct rhetorical possibilities’ (Frasca, 2004, pp. 221–222).

Given the focus on processes in these definitions of games, it is important to recognise that rules are abstractions, and, therefore, necessarily simplifications of complex realities. As McCarty (2004) puts it, ‘a model is by nature a simplified and therefore fictional or idealized representation, often taking a rough and ready form: hence the term “tinker toy” model from physics, accurately suggesting play, relative crudity, and heuristic purpose.’ The distillation, or slicing, of realities (be they historical or hypothetical) is crucial to the use of game design as research, and to research design more generally. Recognising, and embracing, this aspect of their craft, designers must work to identify not only the subset of reality that they wish to model but also realise the assumptions with which they approach that reality. This is where our practices of making, hacking, and jamming are important since they (re)open both the fiction and mechanics of games to contingency, encouraging critique and interrogation. In this we move away from Crawford’s notion of the game as a perfectly ‘closed system’ that must cover all contingencies, and consider a more open concept of the game that promotes critical literacy and an action-oriented research practice that remains open and reflexive about the process of research itself.

### 2.3. From Black Box to Cardboard Box: The Use of Board Games in Our Practice

The ‘open’ concept of the game, where rules and fictions are available to interrogation, and hacking, leads us to work, in the main, with analogue rather than video games. Analogue games include board games, card games, roleplaying games and other forms of tabletop gaming that use physical components. Analogue games have two key affordances that make them a distinct proposition for our research method as opposed to their digital counterparts, namely the visibility and accessibility of their rules and mechanics. Where the immediacy of contemporary video games allows for a photo-realistic audio-visual experience that excels at generating immersive gameplay, board games, in which the interface is more obviously present, offer, deliberately or not, a ‘hypermediated’ experience in which the medium is foregrounded (Bolter & Grusin, 1998). While this comment applies to all the material components of tabletop games (Germaine & Wake, 2024; Wake, 2019; Wasserman, 2020), allowing for interrogation of the whole design process, it is the availability of the rules that makes analogue games most apt for the promotion of critical literacy and action research methods. Gameplay almost always requires the learning of the rules of the game, and, usually, players must manually ‘run’ the game. Conversely, in video gaming, the algorithms controlling the game environment can be largely concealed with the effect that, as Sebastian Deterding has put it, ‘[t]he player stops functioning as a “game executor” and can focus instead on her role as “game player”’ (Deterding, 2009, p. 34). This means that players of board games must engage with a game’s ‘operational rules’ (the rulebook) and many, particularly those playing at a competitive level, will also engage with the ‘constitutive rules’ (the logical and mathematical principles) underlying those operational rules. Taking the role of ‘game executor’ comes with advantages over focusing solely on one’s role as a game player. As Sabin puts it, running a tabletop game ‘obviously requires a lot more intellectual effort from users, just as playing a piece of sheet music requires more effort and understanding than playing a CD, but it also makes the dynamics of the process much more open and explicit, giving users the scope to add their own interpretations and improvisations, and in the end perhaps to produce entire compositions of their own’ (Sabin, 2012, p. 26). Thus, our turn to analogue games arises from the fact that they make explicit the dynamics at work in play structures, opening the rules, mechanics and systemic elements of the game to interrogation and challenge. Just as systems and power structures that determine players’ lives outside of the game are constructed, typically to the benefit of some and disadvantage of others, so are game systems deliberately constructed and enacted in certain ways. If critical literacy and action research alike wish to support people in resisting systematic oppression through critical and collective inquiry, reflection and action (Cammarota & Fine, 2008), board games are a site where this work can take place, typically without resistance, either from a pre-coded game system put in place by developers, or by those with vested interests in the status quo, since board game play is typically seen as a harmless activity, even beneficial for learning.

## 3. Results: Our Case Studies

In the case studies that follow we examine the development of our praxis of game making, hacking, and jamming through results as they manifested in specific examples of our teaching and research. The first case study focuses on teaching practice. In this project, students at the Manchester School of Architecture were tasked with exploring questions relating to Britain’s post-war power infrastructures through the creation of games. Here, game making was about demonstrating learning outcomes and sharing these with a wider (public) audience. The second case study concerns our own research practice in which, recognising the potential of games to produce new thinking rather than reproduce and remediate knowledge, we developed the method of game hacking to investigate research questions. Our third case study details an attempt to make use of these methods in a participatory action research project, which set out to explore how board game play could support young people’s climate action. There, game hacking became an anarchic process that we came to call jamming. These projects demonstrate the critical and radical potential of games as well as the participatory and entangled nature of teaching, learning, research and dissemination, or communication, of that research.

### 3.1. Case Study One: Landscapes of Postwar Infrastructure

Our first case study describes an ‘Appendix’ project attached to the third-year undergraduate unit ‘Landscapes of Postwar Infrastructure’ led by Richard Brook and Luca Csepely-Knorr at Manchester Metropolitan University. The unit, delivered alongside an AHRC-funded project of the same name (which ran from 2020–2022), introduced students to the role of design professionals working alongside engineers in the delivery of large-scale schemes such as motorways, power stations, reservoirs, telecoms, and forestry in the period between 1945 and 1980. In addressing this topic, the unit had the following learning outcomes:

1. Increase your awareness of the history and theory of architecture and landscape architecture in the 20th century.
2. Better understand contemporary practices and collaborations through the understanding of the development of professions, institutions and policies.
3. Develop critical and analytical skills needed to understand the socio-political context in which architecture practice operates.
4. Develop research, writing and discussing/debating skills, constructing an argument, and initiating and sustaining discussions
5. Develop and understanding of the basics of game design and communicating complex research questions through the medium of games to a variety of public audiences

The unit was delivered in the form of lectures and seminars, with learning outcomes assessed by a traditional academic essay.

Alongside the relatively conventional methods of delivery and assessment, the course structure allowed for an additional 5-credit ‘Appendix’ in which staff and students were encouraged to explore alternative learning strategies. The brief for the Appendix, created by Paul Wake and professional game designer Matteo Menapace, tasked students with working in teams to design board games that explored Britain’s post war infrastructure and landscape. These games were assessed against four criteria: ‘Depth’ (a consideration of the design process that asked how far students had gone from repurposing existing games to original work), ‘Information Design’ (the language of the components and rulebook); ‘Metaphors and Mechanics’ (the game’s success in modelling real-world events/systems); and ‘Teachable Potential’ (the extent to which the game functioned as a learning/teaching tool). While the work undertaken for the Appendix supported students in achieving the unit’s learning outcomes, it had the supplementary (and complementary) aims of: (i) developing a practical understanding of the basics of game design (including developing analytical skills through the deconstruction existing games, and information design skills); (ii) prototyping skills (in particular participatory research and observation); and (iii) skills in communicating complex research questions to a variety of publics (in this case, young people aged between 11 and 17).

Students designed their games over a period of six weeks. This work was scaffolded by a series of workshops on game design with Wake and Menapace that ran concurrently with the seminars and lectures on the ‘Landscapes of Postwar Infrastructure’ unit. The resulting games were visually impressive but required further work to develop the rules and overall playability. As Brook, the unit leader reflected in a report delivered to the AHRC, ‘games design is perhaps more of a complex task than we imagined’ (Brook, 2022).

**Figure 1:**
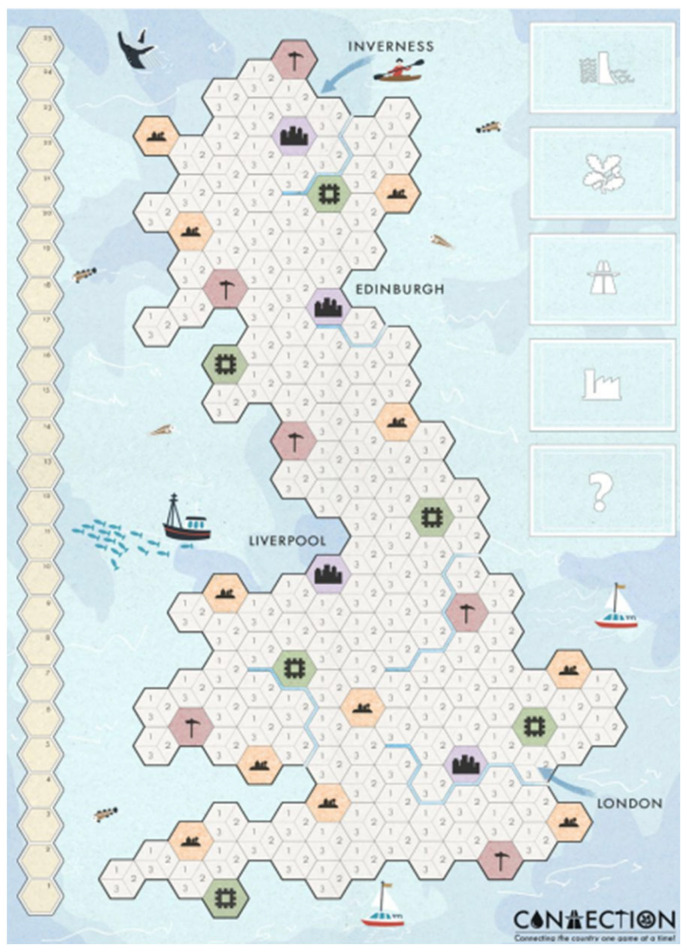
*Connection*.

Two games, *Connection* (a game exploring the connections of Britain’s cities and green spaces with transport and power—see Figure 1) and *New Town Power* (a town-building game that focusses on the provision of utilities and transport links) were taken through several further rounds of playtesting by a group of students and, ultimately, after months of iterative design, were used to share our research with young players, finding audiences at a local secondary school, at the Manchester ESRC Festival of Social Science, and at the Bluedot science and music festival at Jodrell Bank Observatory in Cheshire.

The relative success of the games in engaging various publics with live research notwithstanding, the games made by the students represented a remediation of knowledge, not necessarily a critical interrogation of the production of knowledge. The practice of game making as learning developed through this project mirrors the process as described by Sabin in that it promoted literacy about systems (including social, mechanical and historical systems). However, the focus on making playable games that would communicate digestible lessons, both for the makers and the intended public audiences, did not foster critical literacy of the type we describe in the introduction. Put simply, games gave learners and educators in the project a new mode through which to share their ideas, but not new ideas.

### 3.2. Interlude: Theorising Hacking as Playing with, Not by, the Rules

Encouraged by the successes of game making in the classroom in dialogue with live research, and conscious of the limitations of that work, we set out to explore the affordances of games in our own research. Inspired by the work of activist game makers such as Paolo Pedercini (Molleindustria Games) and Matteo Menapace (Beesness Games), whose games, often made in collaboration with others and in non-academic spaces, offer powerful critiques of the world around us (Menapace, 2017; Pedercini, 2012), our work began from a belief that thinking-with-games might promote more than the re-making of existing models of the world. To this we brought a somewhat contrary-wise approach to rules in games. Specifically, we responded to Bernard Suits’ suggestion that ‘to play a game is to engage in activity directed towards bringing about a specific state of affairs, using only means permitted by rules’ (Suits, 2014, p. 36). To our thinking, the constraints Suits identifies as central to games imply the possibility that alternative actions might be taken. This contention is based on a well-known adage in the discipline in which we both trained (literary and critical theory): rules always imply their own transgression, the imposition of a limit always pre-suppose its crossing (Bakhtin, 1984; Derrida, 1993; Stallybrass & White, 1986). Games, then, hold the promise of anarchic and radical forms of play that question the relation between games (as sets of rules) and players (who are expected to submit to, and obey, those rules). In line with Suits, we acknowledge that such a louche attitude towards rules (accepting them ‘just because’ but also acknowledging that they may be broken at any time) might indeed entail the collapse of the game. It might, though, also entail the arrival of something new. We contend that the anarchic (playful) potential of games, paradoxically made apparent by the prominent position afforded to rules in almost all definitions, and in practice, is key to the kinds of radical, critical, and socially transformative engagement imagined by play scholars, social justice educators, and others seeking to deploy games in radical ways that might challenge hierarchies and power structures. We call this approach ‘hacking.’

What do we mean by hacking? On a practical level, hacking comprises anything from replacing a game’s art and components, changing player counts or roles, shifting from collaborative to competitive dynamics (or vice versa), rewriting rules, and changing, or even abandoning altogether, win and loss conditions (Germaine & Wake, 2024). In this sense, hacking is a tried and tested method, and one that is regularly used by amateur and commercial game designers alike, offering a way of making games quickly by taking something that already exists—likely something that already works—and changing it to a greater or lesser extent. Understood in this practical sense, hacking might describe the way in which Elizabeth Hargrave’s award-winning *Wingspan* (Hargrave, 2019), a game about discovering and attracting birds, might be become a game about dragons, *Wyrmspan* (Vogelmann, 2024), or fish, *Finspan* (Gordon & O’Connell, 2025), or indeed Pokémon, as the fan-made *Pokéspan* (trmptdrummer, 2021) demonstrates. It would be easy to give further examples from other game franchises, but these variants of *Wingspan* are sufficient to illustrate that while reskins use the practical design methods that underpin hacking, the final outputs evidence commercial rather than critical intent, and might be taken as suggesting that there is nothing inherently critical (or subversive) about the practice of hacking games. Indeed, as Soderman (2021, pp. 206–207) has cautioned in relation to indie game jams, such hacking might be seen as ‘playfulness tamed’, putting creative efforts in service of generating novelty that can be commodified, creating games as consumer products. The ‘reskins’ of *Wingspan*, are perhaps better described as ‘remaking’ or, to use a term familiar in game design, ‘modding’ rather than ‘hacking’, suggesting that ‘hacking’, if it is to work in concert with games’ potential to generate opportunities for critical literacy, has a more specific meaning.

This notwithstanding, there is considerable overlap between modding and hacking: both take existing games in new, and perhaps unexpected, directions. The distinction between these practices is not always clear-cut, and individual projects may blur the boundaries between them. However, for our purposes, articulating the difference between modding and hacking is methodologically important because it clarifies our research intentions and the kind of critical work we aim to perform.

Stefan Werning’s concept of ‘ecomodding’ offers a useful entry point for understanding this distinction. Werning (2021) describes ecocritical modifications of commercial games made by fan communities as part of an ongoing discursive process that works across and within established game franchises. He describes modding as a practice that engages critically with game content while largely preserving the procedural systems and mechanics that define gameplay. Following Werning, we therefore understand modding as a practice that works with and within game systems, creating something new while preserving the core mechanics that define how the game operates. In examples given by Werning (2021), mods adapted that system for a new use (such as climate communication). Hacking, as we conceive it, is a more fundamental challenge to the system itself. Where modding accepts the premises of the game and builds upon them, hacking questions those premises and makes visible the ideologies embedded within the system’s structure.

Timo Fluitsma et al.’s (2024) description of their eco-mods of *Magic: The Gathering* as ‘franchise hacking’ helpfully illustrates both the proximity of modding and hacking and the point of difference. They are inspired by our own hacking method to develop the concept of ‘franchise hacking’, which, they explain, ‘seeks to hijack the core mechanics of a franchise to narrate fresh stories, engaging deeply with the game’s language—a language that the fan base comprehends and appreciates intimately’ (Fluitsma et al., 2024). It is this commitment to the game’s core mechanics and vernacular (the ‘franchise’) that aligns this activity with what we would call modding. The slippage between the terms modding and hacking becomes apparent when these newly made games explore how *Magic* itself uses land and resources in relation to conflict. At this point, where the mods turn their attention to the game itself, the practice begins to move from modification toward critical interrogation, from working within the system toward exposing its ideological foundations.

In practical terms, the distinction might be understood as follows: a mod of *Wingspan* that replaces birds with dragons (as in *Wyrmspan*) preserves (and leaves unquestioned) the game’s engine-building mechanics and competitive structure. This is modding. Hacking, by contrast, deliberately exposes the underlying system, making visible, for example, logics of resource extraction and territorial control nature games often obscure with pastoral and cosy themes. This is hacking as critical practice.

We acknowledge that this distinction between modding and hacking exists on a spectrum rather than as a binary opposition. Some practices may begin as mods and evolve into hacks as they become more critically engaged with systemic questions. What matters for our methodology is the effect: does the practice primarily create novelty within accepted parameters (modding), or does it interrogate and denaturalize those parameters themselves (hacking)? Our use of the term ‘hacking’ signals our commitment to the latter—to a critical practice that reads games as ideological systems and speaks back to the worldviews they embed and naturalise.

Our work on hacking diverges from existing commercial (re)making practices to become a form of ‘reading’ the world and speaking back. However, this remains a continuation of its more widespread meaning in popular culture. Hacking began as a practice in the newly-established computer science and engineering labs at MIT in the 1960s and 1970s, as students turned to tinkering with technology to find creative solutions to problems. The definition of hacking in popular accounts of hacker culture since this time, especially since the 1980s and 1990s, describes it as the unauthorised use of, or entry into, a computer system. It has also been described more positively as a mode of creative, hands-on problem solving (McGonigal, 2012, p. 187). More recent scholarly work on hacking co-opt it into the service of neoliberal ideas of productivity and innovation, where ‘hacking’ the system is about finding ways to generate economic growth (Rayner, 2018). The shift in the understanding of hacking as tinkering, largely by students on the MIT programmes of the 1960s and 1970s, to that of hacking as an unauthorised and disruptive intervention, to that of hacking as an entrepreneurial practice can be understood alongside the proliferation of the technologies of the Information Age in everyday life and changing attitudes to technology in the public imagination and among the political establishment. Commenting on the shifting perspectives of ‘hacker culture’, Douglas Thomas states that hacking ought to be understood as ‘more about the imagination, the creative uses of technology, and our ability to comment on culture than about any tool, computer, or mechanism’ (Thomas, 2002, p. 5). This sense of hacking as critical commentary underpins the process that we began to develop in our teaching and research practice, a process which reads game texts through the relation that inheres between the original game text (what might, following Genette’s (1997) work in literary theory, be called the hypotext) and the newly hacked game (the hypertext).

However, our own method of hacking is alert to the tensions and to the ambivalence in the concept of hacking as something unlawful, even destructive, as well as something generative and creative. To avoid co-option back into the systems, structures and contexts the practice seeks to critique, the hacking of games ought not, as we argue elsewhere, lead to the production of new games—and certainly not games that can be sold as new products (Germaine & Wake, 2024). Instead, our method evokes something deliberately destructive at the heart of hacking and we note, indeed, the very word ‘hacker’ comes from word ‘knacker,’ meaning one who undertakes the slaughter of horses. At the same time, we present hacking as a creative method aimed at transforming the systems (and the concomitant stories those systems carry) that frame our experience of the world.

### 3.3. Case Study 2: Suppressive Fire

Our exploration of the destructive and productive affordances of hacking saw us work with a number of games, including the classic card game *Happy Families*, which we remade as a game about the ethics of consumption, the children’s fruit collection board game *Orchard* (Farkaschovsky, 1986) that we used to expose the game’s model of fruit farming as a zero sum competition between humans and ‘crop-damaging’ birds, and the strategy board game *Photosynthesis* (Hach, 2017), a tree-themed game that we turned into a game about tank warfare. The latter is the example that we discuss in detail here to explore how hacking developed in our own research practice.

Published to great acclaim in 2017, *Photosynthesis* is a prominent example among a growing number of nature-themed board games available in the commercial, or entertainment, market. These nature, or ‘eco’ games typically utilise existing mechanics popular with strategy and hobby game consumers, and some are ‘reskins’ of popular older games. Our research sought to evaluate what the benefits might be to this proliferation of nature themes among gamers, and to analyse the representations of ‘nature’ these games offered to their growing player base. Our suspicion was that layering nature themes on existing design patterns might perpetuate extractivist ideologies (Chagnon et al., 2022; Gudynas, 2018; McKay & Veltmeyer, 2021) and other damaging sentiments about ‘nature’ (Germaine, 2022a). The research was conducted in preparation for a participatory study we were designing with young people to examine board games as cultural objects in the context of climate change (see below) and for a broader project on the issue of ‘ecogames’ we were developing with colleagues across Europe in preparation for a Horizon-UKRI grant proposal. The game we focused on in our preliminary research is, as its title suggests, about photosynthesis, the process by which plants (in this case trees) convert light energy into chemical energy. It was selected because it was a highly popular and acclaimed example of a nature board game, and one of the early examples of its type.

*Photosynthesis* is a competitive game, each player taking the ‘role’ of a species of tree with the goal of accumulating the greatest number of points by harvesting (removing) their trees from the board, with those closer to the centre of the board having the highest value. Play takes place on a board divided into 37 circular spaces arranged in a hexagonal pattern and representing the forest floor. Players begin by placing two small trees on the outer ring of the board and a sun segment is placed to show which of the trees receive light, and which are in shadow. The game has two phases. In the ‘Photosynthesis Phase’ players collect light points based on the number and size of their trees that are in the sun (which moves around the board each turn, necessitating forward planning to avoid trees falling under the shadow of other trees). In the ‘Life Cycle Phase’ players spend light points to carry out actions: Buying seeds/trees; Planting seeds; Growing trees; and Collecting (scoring points by removing trees from the board). The game ends after eighteen rounds (when the sun has completed three revolutions of the board), at which time the player with the most points (the person who ‘collected’ the most trees) wins.

*Photosynthesis* may have kick-started a new trend for green games, but there is little that is new about its mechanics. The game is an engine builder in which there are two main strategies—prioritising the accumulation of light points by developing a game engine that will produce them an increasing rate, or by blocking others’ accumulation of light points by planting trees that throw shadow onto their trees. Both strategies emphasise area control and the maximisation of profit and return on investment. As Healey (2017) put it in his *Dice Tower* review, *Photosynthesis* is ‘an abstract strategy game with an economy.’ However, we felt that the economy of the game was anything but abstract. The mechanics of area control, resource extraction, accumulation, and domination are those of colonial capitalism, and economy is as much a theme as the pretty trees emphasised on the box art. That the resource over which players compete is sunlight makes the ideologies of the game’s mechanics readily apparent: there is nothing more abundant than sunlight, and nothing natural about a system that would apportion and count sunlight as a finite resource. Moreover, in woodland communities shading is typically a matter of who fares best in which conditions. Taller beech trees shade their young to encourage slow growth, for example. As many ecologists (Beiler et al., 2009; Wohlleben, 2021) have pointed out, the woodland is a site of collaboration not competition. The system underpinning *Photosynthesis* is therefore neither a simulation of an ecosystem nor an abstract ruleset. It is the system of colonial capitalism.

The abstractions of game systems reveal an underlying conception of nature as a Hobbesian state of war, ‘every one against every one’, an ideology that naturalises aggressive competition (Hobbes, 1651, p. 64). These game systems therefore also naturalise the violence on which capital accumulation is founded *and* reiterate a mechanistic vision of nature, which supposedly runs, and can be managed, as a machine. Our point is not that the game system of *Photosynthesis* is a mismatch for its theme, what might be called ‘ludonarrative dissonance’ (Hocking, 2007), though it is the case that contemporary research on woodland ecologies emphasises complex forms of collaboration rather than the survival of the fittest. Rather, we suggest that game’s aesthetics, which include its formal elements (in other words both the ‘ludo’ and the ‘narrative’), reveal a war-like story about ‘nature’ that has underwritten and sought to naturalise centuries of capitalist production and accumulation. As Germaine (2022a) wrote in an early analysis of the game, *Photosynthesis* ‘operates on the logic of wargames’ and could ‘easily be re-skinned such that its tree avatars were represented by military units.’

With this critique in mind, we decided to make ‘*Photosynthesis: The wargame*,’ setting the game in World War Two, during the Battle of the Bulge (16 December 1944–25 January 1945), to take advantage of one of the most immediately recognisable global conflicts. We wanted the hack to remain as close to *Photosynthesis* as possible. In effect we would ‘reskin’ the game with the intention that the original game system would be immediately apparent. Our intention was not to make a wargame proper and as such we made no pretence of working towards a detailed (playable) historical simulation of the relief of Bastogne. The potential discomfort of this is clear: this is ‘dark play’ (Schechner, 2002), designed to make players feel uncomfortable and unsettled. With the historical aspect, and therefore the educational and/or training value, of the wargame stripped away, and military conflict placed over the abstract game as a ‘marketing’ or ‘advertising’ gimmick a game of this kind might simply be seen as capitalising on war’s longstanding appeal in consumable media. For us, though, dark play is a potentially productive space, capable of disrupting stable and unthinking routine and prompting the kind of reflection required in critical literacy. The discomfort of playing at war without the ameliorating effects of simulation or training is something we hoped would provoke reaction and reflection on the part of players who might note that games (classically defined) have conflict at their core and that *Photosynthesis*, despite its appealing artwork, similarly has conflict at its heart. That the hack reveals this was confirmed at a workshop we ran as part of the Games Transformed festival in 2024 when one participant remarked that the game was ‘not so much a reskin as a deskin.’

**Figure 2:**
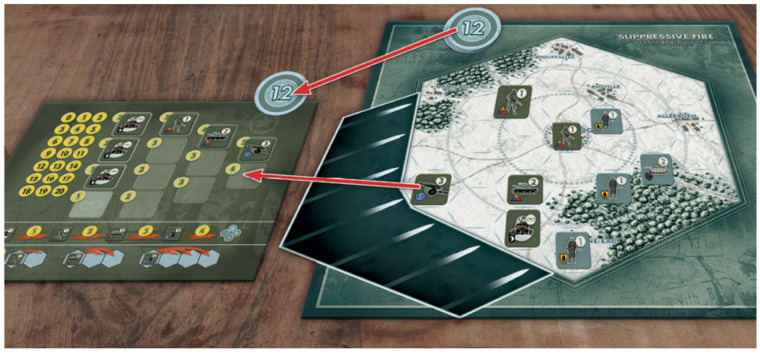
*Suppressive Fire* player board, game board and directional template as designed by Marc von Martial for the authors.

Accordingly, the rules of the game we made remain the same as the original, the only exception being that we determined that our version would be a two-player game to emphasise the combative nature of the original. Our actions were therefore limited to the renaming the various game mechanics and reflecting these changes with new artwork. We recruited Marc von Martial, a game artist with a track record of wargames design, to produce the board, tokens and rulebook, replacing all in-game assets with equivalents that evoked the aesthetics of traditional manual wargaming (hex grids, square die-cut tokens, recognisable historical units). The result was *Suppressive Fire*, a game in which seeds become military scouts, seedlings infantry, medium trees tanks, and large trees artillery (see Figure 2). What had once been the shadows cast by trees, was now the area subject to the suppressive fire of these various military units, a change from which the game took its new name. The rotation of the sun becomes the direction of suppressive fire, which prevents units from moving in certain directions and occupying useful vantage points. The woodland map itself became a battlefield, with the most valuable ‘soil’ replaced by strategically valuable locations. The ‘light point’ economy became a military economy, allowing players to purchase more units for deployment.

Our de-skin of *Photosynthesis* into *Suppressive Fire* takes the notion of competition in nature to its terrible conclusion: a war of all against all in which collaboration is not possible. In contrast, the messy lives of real trees, characterised by inter-species symbiosis, parasitism, collaboration and relationships of all kinds, suggest an altogether more complex reality, one that cannot easily be captured by formal abstractions, nor reduced to a collection of stocks of resources nor to a background environment or terrain—as in traditional war and hobby games alike.

In this example, which differs from the *Wingspan* rethemes we mention above as examples of commercial modding, re-skinning came to be understood by us as a way of de-naturalising systems that are typically presented as seemingly ‘natural.’ Reskinning *Photosynthesis*, in this sense revealed that what was presented to players as a neutral biochemical and ecosystemic process (photosynthesis and the expansion of tree species through seed dispersal), was in fact a story about economics and territory control. Our hacking practice in this early stage of our research stops short of versions of critical play that openly disrupt or disobey rules, leading to emergent phenomena arising from the possibilities endowed by play but not necessarily acknowledged within formal rulesets and mechanics (Flanagan, 2009, p. 60). This is because we were interested in reflecting upon the rules specifically in our research process. The rule-breaking mode of critical play Flanagan (2009, p. 61) describes, in contrast, implies the possibility of system change, of ‘moving and playing within structures and systems to create an ‘open environment focused on experimentation and subversion.’ Although she accepts Crawford’s idea of the game as a closed system, with cohesive and integrated elements, and suggests that it is not always possible to be free of the rules, Flanagan suggests that game designers can encourage critical play by working ‘like a virus from within to infect and radically change what is expected and what is possible when players play’ (Flanagan, 2009, pp. 61–62). Despite refusing to break the rules, and, indeed, keeping every single rule of the game intact, this viral effect is precisely what our *Photosynthesis* hack enables. The process of hacking as re- (or de-)skinning, deployed in service of a critical literacy, can dramatically reorient players in terms of their relationship to the gameplay, radically altering how we read the original game and inviting critical reflection.

### 3.4. Case Study Three: Hacking in Action Research and the Turn to Jamming

In this final case study, we describe the Game in Lab/Libellud Foundation funded project ‘Games Imagining the Future: Play and the Environment’ to consider how game hacking intersects with the tenets of Youth Participatory Action Research (YPAR), thereby making explicit how our hacking practice is a form of critical literacy in the tradition of radical pedagogies on which participatory action research is founded. The project, which took place between 2021 and 2022 in Greater Manchester, set out to investigate how board games might be used to support young people’s understandings of the climate crisis; to evaluate board games as a tool through which young people can explore and share their ideas about climate change; and to identify the ways in which games mobilise individual or collective action. Additionally, we sought the expertise and viewpoints of young people on the design patterns and trends we saw proliferating across the board game industry.

Our initial proposal described a series of ‘playing and hacking’ workshops in which people would identify themes that were important to them as a group, before deconstructing and remaking games to exploring those ideas. Before carrying out the participatory work, we imagined giving participants games to break and remake as they wished, transforming off-the-shelf board games into new playful experiences promoting the young participants’ visions of climate futures. The expected outputs included a co-authored academic paper, a toolkit for designers co-produced with young participants, and an annotated ludography of ‘Eco Games’ containing reflections written by young participants.

**Figure 3:**
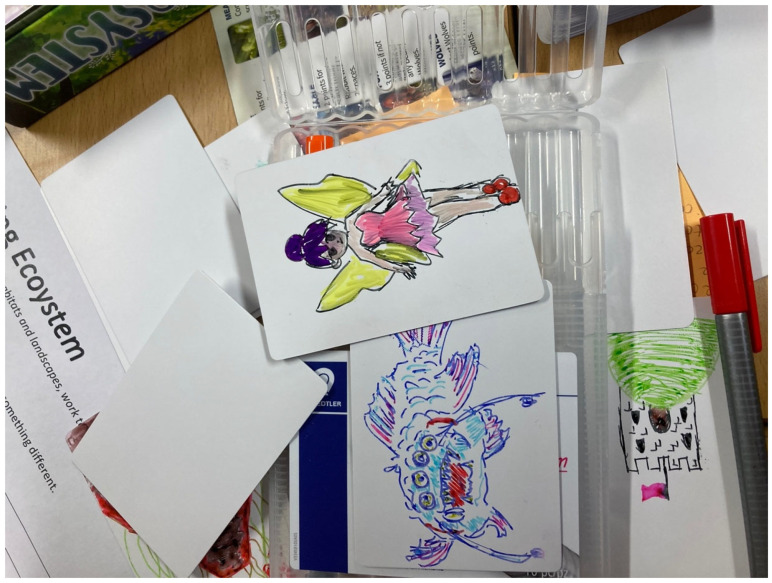
Characters Created by Participants for Matt Simpson’s card game, *Ecosystem* (Simpson, 2019).

**Figure 4:**
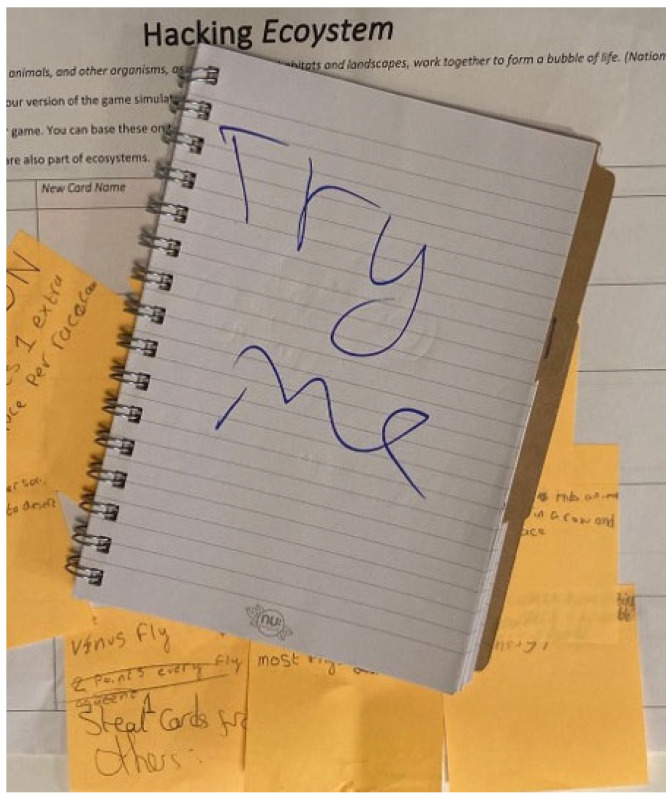
A notebook from the sessions which participants often used to challenge one another’s ideas for new game rules.

The project did not go as planned, though it far exceeded our expectations. While in our project design we described hacking using terms like ‘mangling’ and ‘mayhem,’ the hacking sessions were far more disruptive than we had envisaged in our original research design. The sessions unfolded into messy chaos in which students ate cake, doodled, wrote notes, made pictures, hoarded, drew on, and played with game components in ways that the original designers never intended (see Figure 3 and Figure 4). The sessions, which are described in detail in a forthcoming co-authored paper written by ourselves and university student facilitators (Germaine et al., Forthcoming), were unproductive according to the criteria of doing research since most of our intended outcomes never materialised, and not a single game the students made in the hacking sessions could be replicated beyond the live sessions.

Rather than being productive of a design toolkit, or new, climate-conscious games, hacking became a way for participants to reflect on issues such as their perceived powerlessness in society, social conflict, and the seemingly inevitable inequalities that drive crisis. As participants ourselves within the research workshops, we reflected on the same issues alongside the young people. At the same time, as researchers engaged in participatory action research and conscious of funder expectations, of promises we had made regarding various publishable outputs, we reflected on an appropriate and ultimately invigorating powerlessness. As unicorns and hellsnails were added to serious games about planning ecosystems, and complex and ever-evolving roleplaying games emerged through the weekly sessions, we realised we ought not to anticipate outcomes for our sessions and that providing structure to the hacking both invited and deserved the irreverence with which the participants responded.

These reflections on the unproductive nature of the project prompt deeper thought on the process (and outcomes) of research. In designing the project, we followed the tenets of YPAR and participatory practice in aiming for ‘various degrees of collaboration’ between ourselves and our participants, and we understand that YPAR ‘involves all participants being free to challenge the thinking and practice of others’ (Percy-Smith et al., 2019, p. 191). This challenge to our research design and its adherence to the ends-in-advance agenda of academic research occurred early in the project. As the workshops developed, we had to rethink not only what our outcomes would be, but also our concept of hacking. Moving away from definitions of hacking that relate to creating new things, and even from ideas of hacking found in spy movies, cybersecurity, or that imply ‘breaking into’ computer systems, we approached a concept of hacking that was both messy and intimate, one that dismantles rules and systems, and allows us to explore new worlds. Such worlds might reflect the hackers’ own or may imagine something completely new and excessive.

What we learned during our sessions with our young participants was that the intimate and messy process of hacking may, ultimately, act as a blockage in production, rather than as a point of creation. As one of our student facilitators put it, ‘the students enjoyed pushing back against the rule setter, regardless of how futile and unfair it appeared, right up to the point where the game was packed away. To not win or lose, but to simply stretch out the game creation process to ignore the finality of the game’s end’ (Germaine et al., Forthcoming). Following this reflection, we consider game hacking as akin to ‘culture jamming,’ a radical action that forces a change in direction through a refusal to participate according to the rules. We came to call this practice, the practice of inhabiting the moment of stalled production, ‘jamming.’

## 4. Discussion of Results: Jamming

Our work on the ‘Games Imagining the Future’ project led us to consider the relation between hacking and jamming. Before explicating our use of the term, however, we must acknowledge that ‘jamming’ already describes an established method within game development practice. Game jams are typically time-bound events where participants rapidly prototype games around specific themes or constraints. They have become a staple of game development culture and are beginning to be deployed for critical purposes. The Bristol Digital Game Lab’s ‘concept’ game jams, for instance, demonstrate how the format can be directed toward ‘critically examining complex problems’ (Bristol Digital Game Lab Events, n.d.). These events leverage the creative energy of traditional game jams while orienting them toward conceptual inquiry rather than commercial viability. Our use of the term ‘jamming’ acknowledges this emerging critical practice but also seeks to reorient the notion of jamming toward something more radically unproductive. Where concept game jams repurpose the game jam format to generate critical prototypes and provocations, our notion of jamming moves further from outcomes and deliverables altogether. We appropriate and re-inflect the term to describe not a method for making games differently, but a practice of refusing to make games in ways that serve predetermined agendas.

We came to see our own project’s title as prophetic, drawing attention as it does to the fixation on futures (outcomes, results, end products) over present processes (imagining). The time spent with the young participants in our weekly sessions gave us a renewed appreciation for the slowness required for game making as a critical practice. In this our work aligns with the call for slow research (Davidson, 2025; O’Neill, 2014; Ulmer, 2017) with its focus not on swift prototyping and output, but on reflection, creativity, and collaboration. ‘In this sense *slow* is a catalyst for conducting inter-disciplinary conversations and critical research that may disrupt, lead us to think deeply and critically. There is something in the idea of *slow* that is a call to thinking otherwise, for thinking dialectically’ (O’Neill, 2014, p. 201). We relate the call for slow research and ‘thinking otherwise’ to the radical pedagogical traditions evoked in the introduction to this article, considering slow research (and unproductive sessions such as those we took part in with our young participants) as ‘counterfoil’ research (Illich, 1973), and the ‘continuing, hopeful enquiry human beings pursue’ with one another, and through critical reflection (Freire, 2017, p. 45).

To return to the example of *Suppressive Fire* in the light of these reflections, we ask what was our purpose in making the physical game? This was a process that took a great deal of time and effort, including liaising with a professional designer to produce a game that looked something akin to the commercial game it was hacking. In this process, the ‘final’ version of *Suppressive Fire* was not the end of the research, but only a prompt to further critical reflection and thinking, which we did with various players at workshops, demos and festivals. This process follows Freire’s conception of critical literacy in its equal share of effort given to reflection and action. Similarly, it represents a shift from the concrete (an existing game that represents how ideologies are perpetuated by cultural objects) to the abstract (a reflective analysis of the game’s mechanics and dynamics) back to the concrete (a transformed gameplay experience that gave rise to new reflections for those who engaged with it). In this, our hacking as research practice follows the dialectical process outlined by Freire (2017, p. 78), in which concrete situations are never reduced wholly to the abstract, but in which reflection and action allow for a ‘critical perception’ of concrete realities. We see a similar, perhaps more radical, potential, in hacking as it teeters on the edge of jamming.

Our thinking about jamming started with the idea of game jams. A staple of both informal and formal work on games, game jams are events in which people come together to design games (usually video games) from scratch. They are often time bound (lasting on average 48–72 h), sometimes location bound, often themed, sometimes competitive and sometimes not. While there is sometimes a home-spun and anti-corporate aspect to game jams, they are often bound up with existing industry practices, and the wild innovation that they encourage often serves to generate new products and to identify new talent. Focused on the generation of reproducible outcomes at speed, these were not the jams we were looking for.

Our approach to jamming comes from a different angle. Recalling our experience with young research participants in which jamming, while wildly creative, was, seemingly, also productive of nothing. Our experience there recalled to mind Caillois’s (2001, pp. 9–10) description of play as ‘unproductive’: ‘Nothing’, he writes, ‘has been harvested or manufactured, no masterpiece has been created, no capital has accrued. Play is an occasion of pure waste: waste of time, energy, ingenuity, skill.’ The jamming that our young participants engaged in was, however, anything but a waste of time. It combined the jazz inspired sense of jamming with the sense of jamming as in being stuck fast, as ‘the blocking or stopping of a machine’ (*OED*). Perhaps inspired by the nature-themed games with which we worked, our attention was caught by the *OED*’s many entries on jamming entries relating to logging, specifically to log-jams, a phrase that is both literal and figurative.

In its figurative sense the ‘log jam’ has negative connotations, it is unproductive, it refers to things that stop progress. However, literal log jams suggest something more positive. We take our cue from the natural world in which log jams, in frustrating the flow of rivers, are generative rather than restrictive. In the world’s largest recorded log jam, the 51 square kilometre woody deposit in the Mackenzie River Delta in Nunavut, Canada, for example, fallen logs store around 3.1 million metric tons of carbon (Sendrowski et al., 2023). Meanwhile, so-called ‘engineered log jams’ are used for bank protection and habitat enhancement, providing a sustainable approach to river management. Natural or engineered, ‘in the form of overhanging logs, rootwads, and especially logjams, large woody debris creates pools, partitions habitat, and provides complex cover. Pools form around any material that creates friction and resists displacement by flowing water’ (Herrera Environmental Consultants, 2006, p. 15). This friction, a resistance to flow, is at the heart of jamming as a critical and radical practice.

Flow, understood as a state of complete absorption during which one might not even note the passing of time (Csikszentmihalyi, 1975), is at odds with the kinds of critical positioning that might inform transformative reflection and action. Indeed, as Soderman (2021, pp. 5–6) suggests, flow privileges individualism over social collectives, growth and accumulation over equilibrium and sustainability, self-determination over the idea that external forces shape human consciousness, and action over critical examination. Flowing subjects are not simply game players experiencing the psychological state of flow; they are being positioned as media consumers in a way that promotes flow’s ideologies.

In other words, if games as consumable products aim to evoke flow, they run the risk of promoting passivity and capitulation to the status quo. In response to these challenges to the disruptive potential of games, but not entirely abandoning the optimistic sense of the playful, which Sicart (2014, p. 22) describes as an *attitude* in contradistinction to the *activity* of play (Sicart, 2014, p. 22), we find in jamming, rather than in playing, making or hacking games, a means for disrupting the logic of consumption and conformity that characterise games as a media that serve hegemonic power structures. We suggest that jamming, as an extreme form of our hacking practice, is a much-needed disruption of such passivity, and a way of engaging with games that shifts agency from the dominant system that the game models or promotes, returning players to the point at which the rules and system of the game might be (re)opened to negotiation.

The friction created by jamming is not an endpoint but a pause within an ongoing cycle of critical engagement. This friction, experienced as a refusal to flow smoothly to predetermined outcomes, generates conditions for the deeper reflection and consciousness raising that Freire (2017) identifies as essential to transformative action. However, we wish to guard against the impulse to instrumentalize jamming as preparatory to some more productive phase. The goal may be to remain within the jam, and to resist the pressures, institutional or otherwise, for resolution and product. The log jam metaphor is productive precisely because it creates habitat, stores carbon and enriches the ecosystem. Staying jammed is the radical practice. Jamming as research practice is valuable precisely because it disrupts the extractive logic that treats inquiry as a process of harvesting outputs. It therefore works against what Freire (2017, p. 45) describes as the negation ‘of education and knowledge as processes of inquiry’ seen in models of education that emphasise the depositing of knowledge and training of citizens to become part of a productive apparatus.

Finally, we would suggest that the relationship between jamming and radical game-making is not linear but recursive. Our experiences suggest that cycles of making, hacking, and jamming inform, and may bleed into, one another. Using game making in the HE classroom led us to employ hacking in our desk-based research, which in turn prepared us to recognise and value the anarchic jamming that emerged in our participatory project with young people. That jamming experience has subsequently transformed how we approach both teaching and research, making us more attentive to moments when participants (and ourselves) might be more willing to dwell in apparent unproductivity. We find ourselves returning to making and hacking with a different orientation, more attuned to the potential for games to hold open questions rather than deliver answers.

For educators and researchers drawn to games as tools for critical pedagogy and transformative research, we therefore offer not a neat methodological sequence but an invitation to embrace the productive tensions between making, hacking, and jamming. The critical practice we suggest here is one that remains reflexive about its own processes, attentive to power dynamics, and committed to the slow, collaborative work of building critical literacy, especially when that work resists tidy conclusions. If jamming creates spaces where participants can interrogate power, imagine alternatives, and practice collective refusal, then perhaps the radical practice is learning to sustain the jam and to remain in the generative discomfort of questions not yet answered and games not yet finished.

## Figures and Tables

**Table 1:** From making to hacking to jamming.

Approach	Definition	Application	Example
Making	Creating games as a learning outcome, remediating existing knowledge into playful forms.	Allows students to demonstrate learning through design; communicates complex ideas to diverse audiences; shares research with publics; remediation rather than production of new knowledge.	Case Study 1: Landscapes of Postwar Infrastructure
Hacking	Interrogating and transforming existing games to reveal embedded ideologies and speaking back to the world.	Fosters critical literacy; functions as both analytical method and creative intervention; aligns with action research practice.	Case Study 2: *Photosynthesis*
Jamming	An anarchic, reflexive practice resisting productivity and predetermined outcomes.	Democratises research by refusing fixed hierarchies; values process over product and emergence over deliverables; embraces “unproductive” time as generative.	Case Study 3: Games Imagining the Future

## Data Availability

The original contributions presented in this study are included in the article. Further inquiries can be directed to the corresponding author.
